# Supplementary material for: Are carbon emissions trading and green financial instruments synergistic? -Comprehensive quantitative research based on content analysis
Source: PLoS One. 2024 Mar 7;19(3):e0298601. doi: 10.1371/journal.pone.0298601 (PMC10919601; doi:10.1371/journal.pone.0298601)
Supplement: S1 Data — (ZIP) [file pone.0298601.s001.zip › Data/地级市Z_F工作报告.zip!/─┌├╔╣┼/║⌠┬╫▒┤╢√╩╨/└┤╫╘╣1⁄2╓┌║┼í╛┬φ┐╦╩2╛▌═°í┐_2014.docx]

各位代表：

现在，我代表市人民政府向大会报告工作，请予审议，并请市政协委员和列席会议的同志提出意见。

一、2013年工作回顾

2013年是我市经济社会发展负重前行的一年。在自治区党委、政府和市委的领导下，市政府团结带领全市各族人民，认真贯彻自治区“8337”发展思路和市三次党代会精神，狠抓投资、项目和结构调整，更加注重生态、民生和社会事业，有效克服宏观经济下行影响，全面战胜洪涝等自然灾害，成功迎接自治区推动科学发展现场观摩检查，经济社会保持了稳中有进、稳中向好的发展态势。全市地区生产总值完成1430.6亿元，增长9.5%。固定资产投资完成1080亿元，增长20.1%。地方财政总收入完成151.2亿元，增长10.1%。规模以上工业增加值增长13.2%。社会消费品零售总额完成450亿元，增长12.9%。全面完成了自治区下达的节能节水减排任务。

（一）投资和项目建设持续推进。建立重点项目和重大事项推进机制，出台鼓励扶持工业经济发展的政策措施。全年共实施1835个项目，发展动力进一步增强。神华1000万吨褐煤提质、北方药业二期、阜丰技改等项目试生产，驰宏20万吨铅锌冶炼项目进行调试，宏裕“3052”化肥等项目进展顺利。房屋建筑面积1414万平方米，实施了供热管网、城市道路等286个城镇基础设施项目。牙克石-海拉尔高速公路全线通车，阿荣旗-扎兰屯铁路竣工运行，扎兰屯支线机场和4个通用机场已获批准。扬旗山水库开始蓄水，Z866、扎敦水利枢纽和尼尔基水库下游30万亩灌区等项目开工建设。呼伦贝尔经济技术开发区输变电工程投入使用。

（二）经济结构进一步优化。三次产业结构调整为18.4：47.7：33.9，工业主导地位进一步巩固。工业内部结构不断优化，非资源型产业完成产值830亿元，占工业总产值比重达62%。农牧业内部结构调整加速，粮食产量实现“十连增”，达到120亿斤，荣获全国产粮大市称号。玉米种植面积超过900万亩。牧业年度牲畜存栏1882万头（只），农区牲畜头数保持增长，占牲畜总量的72.8%。制定了促进草原畜牧业转型升级三年行动规划，进一步明确了草原畜牧业发展的方向、重点和措施。组建了呼伦贝尔肉业集团，年屠宰肉牛能力达到10万头。服务业保持良好发展势头，全年共接待游客1140.5万人次，增长13.5%，旅游业总收入完成278.8亿元，增长30.5%。满洲里万达、海拉尔红星美凯龙等商贸综合体开工建设。建立激励约束机制，调动了金融机构支持地方发展的积极性，全年各项存款余额1114.3亿元，增长11.6%；各项贷款余额621.9亿元，增长12.2%。

（三）县域经济和非公经济活力增强。出台了促进县域经济发展的实施意见和非公有制经济中长期发展规划。注重培育特色产业，县域经济发展总体加快，10个旗市规模以上工业增加值增速高于全市平均水平。注重优化发展环境，加大政策支持，全年新增中小企业贷款35.3亿元，占新增贷款的52%。非公经济产值、固定资产投资和吸纳就业占比分别达到51.8%、50.2%和48.8%。

（四）改革开放成效明显。简政放权实现新突破，对645项行政审批事项进行清理，取消、下放、精简422项，占行政审批事项的65.4%。理顺呼伦贝尔中心城区管理体制，新区管委会获得自治区批复。通过了农垦集团组建实施方案，集团改革深入推进。呼伦贝尔经济技术开发区晋升为国家级开发区，赋予其市级管理权限。城投集团和水务投资公司投融资能力明显增强。集体林权制度改革和事业单位分类改革稳步推进，公立医院改革和财政管理改革进展顺利。

我市整体纳入《黑龙江和内蒙古东北部地区沿边开发开放规划》，《满洲里国家重点开发开放试验区建设总体规划》获得国家批复，《呼伦贝尔中俄蒙合作先导区建设实施方案》上报国家待批。口岸基础设施建设有新进展，“苏满欧”专列首次开行。开展了满洲里异地办证、海拉尔航空口岸外国人入境签证和台湾居民入境签注业务。外贸实现逆势增长，口岸过货量突破3000万吨，增长6.5%。外贸进出口总额完成23.7亿美元，增长8.3%。招商引资取得新成效，引进国内（市外）资金791.4亿元，实际利用外资2.2亿美元。“呼伦贝尔——天人合一的地方”城市宣传片亮相纽约时报广场，提升了我市的知名度和美誉度。

（五）生态环境保护与建设不断加强。治理沙地100万亩，禁牧和草畜平衡超过1亿亩，造林绿化120万亩。森林采伐量控制在110万立方米以下。严格落实草原生态保护补奖政策，发放资金5亿元。绿色矿山、和谐矿区建设扎实推进，完成了宝日希勒塌陷区治理。呼伦湖生态恢复成效明显，水位提高1.36米，水面扩大167平方公里。呼伦贝尔自然博物馆开工建设。

（六）保障和改善民生力度加大。全年民生支出222亿元，占财政支出的70.7%。城镇居民人均可支配收入和农牧民人均纯收入完成21482元和9990元，分别增长10.2％和13.4％，均高于GDP增速。深入实施“扶贫攻坚工程”，统筹推动连片开发和整村推进等专项扶贫工程，扎实做好重点帮扶、定点扶贫和领导干部联系贫困旗县苏木乡镇工作，4.9万人实现脱贫。扎实推进“创业就业工程”，全市城镇新增就业3.66万人，高校毕业生就业1.26万人，农牧民转移就业8.4万人，城镇登记失业率为3.85%。高度重视“百姓安居工程”，投资29.78亿元，开工建设44943户，基本建成20973户，均超额完成自治区下达的指标任务。切实加大社会保障力度，城乡居民养老保险制度实现全覆盖。企业退休人员养老金增加到1532元/月，城镇居民基本医疗保险补贴标准提高到280元/年，城镇居民大额补充医疗保险限额提高到6万元。城乡低保人均补助标准分别提高17.1%和16.3%，农村牧区“五保”标准提高12.4%，城镇“三无”人员和孤儿补助分别达到700元/月和1100元/月。为23万户低收入家庭每户发放600元购煤补助。投资11.5亿元，对中心城区热网进行改扩建，新增供热能力1500万平方米。解决了15万人的安全饮水问题。完成万套农户科学储粮设施建设，新增储粮能力1亿斤。实施“菜篮子”工程，新建5025亩蔬菜基地和一批蔬菜储备库，新设55家蔬菜平价店，缓解了菜价上涨压力。

（七）社会事业稳步发展。组团参加了蒙粤科技合作活动周，成立了呼伦贝尔生态产业技术研究院，海拉尔区、牙克石市等6个旗市区获得“全国科技进步考核先进县（市）”称号。各级各类教育协调发展，教育教学质量稳步提高，建成39所幼儿园和38个风雨场馆。市图书馆投入使用,市规划馆、科技馆、艺术馆主体工程竣工，蒙古族源与元朝帝陵综合研究和内蒙古蒙古族源博物馆项目启动，五彩呼伦贝尔合唱团随同习近平主席出访俄罗斯演出，歌舞诗剧《呼伦贝尔大雪原》荣获“文华奖”优秀剧目奖。完成11万户广播电视户户通建设任务。120医疗急救指挥网络建设全面铺开，卫生信息平台及应用系统投入使用，医疗救治体系进一步完善。民族宗教、人口计生、档案史志、体育、规划、气象、人防、工商、地震、工青妇、红十字会、科协、关工委、残联和老龄等工作都有新进展。

（八）抗灾救灾取得全面胜利。去年，我市遭遇了历史罕见的洪涝灾害，54.7万人受灾，直接经济损失25.96亿元。灾害发生后，在自治区党委、政府的关心支持下，我们积极应对，统筹抓好抗灾救灾和灾后重建。拨付资金6亿元，修复水毁房屋12402户、重建1828户，完成了40处水利工程、20.6公里干线公路、1373.2公里农村公路和供暖、供水等基础设施修复工作，近9万受灾户获得保险理赔。下拨7394万元生活补助及帐篷、棉衣、棉被褥等各类生活物资，保证了受灾群众“有饭吃、有水喝、有衣穿、有房住、不受冻”，无一人因灾死亡。

（九）民主法治建设继续加强。认真执行市人大及其常委会决定，自觉接受市政协民主监督，广泛听取各民主党派、工商联和无党派人士意见，全年共办理人大议案1件、建议108件，政协提案218件。严格落实中央八项规定、自治区28项具体要求和市委相关要求，狠抓作风转变，全市“三公”经费压缩24.2%。加强公车管理，封停116辆奥迪车，取消350辆公车免缴车辆通行费特权。党政机关、事业单位办公用房清退工作基本完成。加强政府法制建设，积极推进“六五”普法和政务公开，加强审计监督和社会监督，行政执法和公正司法水平逐步提高。实施“平安呼伦贝尔建设工程”，严厉打击各类违法犯罪活动。开展领导干部大接访，信访总量下降20.4%。安全生产形势平稳，各类事故下降50.1％。加强社会管理，我市被评为全国“社会管理综合治理优秀市”。开展农村牧区社区建设试点工作，全市92%的社区办公活动场所超过300平方米。双拥和边防工作得到加强，承办了全军边境综合防卫管控能力建设研究活动。全市民族团结、社会稳定、边疆安宁的大好局面进一步巩固。

各位代表！过去一年的成绩来之不易。这是在市委领导下，全市各族人民共同奋斗的结果，是市人大、市政协有效监督的结果，是社会各界鼎力支持的结果。在此,我代表市政府，向全市人民，向人大代表、政协委员和各民主党派、人民团体，向驻呼军警部队指战员和所有关心支持呼伦贝尔发展的同志们、朋友们，表示衷心的感谢!

回顾总结过去一年的工作，我们也清醒地认识到发展中还存在许多矛盾和问题：经济总量小，发展不足，加快发展仍是我们的主要任务。经济结构不优，发展方式粗放，可持续发展能力不强，调整经济结构、转变发展方式的任务紧迫而艰巨。我市处于市场末端，大宗工业产品和原材料市场低迷、价格低位，市场约束仍然存在。县域经济发展不平衡，非公经济活力不足，产业支撑能力薄弱，基础设施建设滞后，经济发展自主增长机制尚未形成。基本公共服务不均衡，财政收入放缓，民生支出刚性增长，政府收支矛盾突出。社会建设有待加强，发展环境亟需改善，政府公信力、执行力应进一步提高。部分干部不同程度存在形式主义、官僚主义、享乐主义和奢靡之风，缺乏责任意识和担当意识，抓落实和破解难题的能力需要增强。一些长期积累的影响发展稳定的深层次矛盾和新形势下产生的网络舆情等新问题交替叠加，全面深化改革和加强社会治理任务艰巨。对此，我们要高度重视并切实加以解决。

二、2014年主要任务

2014年是贯彻党的十八届三中全会精神、全面深化改革的开局之年，是深入落实“8337”发展思路的关键一年，也是实现“十二五”规划的攻坚年，做好今年的工作十分重要。

今年政府工作的总体要求是：以党的十八大、十八届三中全会精神、习近平总书记视察内蒙古时的重要讲话为指导，深入贯彻落实自治区“8337”发展思路和市委三届五次全委会暨经济工作会议精神，立足当前保增长、谋划长远调结构、全面改革增活力、扎扎实实惠民生，转方式、强基础、抓城镇、保生态，努力开创美丽发展、科学崛起、共享繁荣的新局面。

今年经济社会发展主要预期目标是：地区生产总值增长9.5%左右，固定资产投资增长16%，公共财政预算收入增长8%，社会消费品零售总额增长13%，城镇居民人均可支配收入增长10%，农牧民人均纯收入增长11%。城镇登记失业率控制在4%以内，人口自然增长率控制在5‰以下，主要约束性指标达到自治区要求。

从今年经济工作面临的大形势和大背景看，这是一个积极的、自加压力的目标，只要我们按照市委的总体要求，以改革的精神、思路和办法，在化解下行压力中坚定信心、尊重规律、因势利导、主动作为，在具体工作中抓住主要矛盾、理顺重大关系、把握关键环节、狠抓工作落实，是能够实现的。

实现上述预期目标，今年重点抓好以下十项工作：

（一）持续加大投资和项目建设，确保经济稳定增长

经济欠发达、发展不足的基本市情决定我市将长期处于建设阶段，建设的核心就是投资和项目。只有抓住这个关键，才能掌握保增长的主动权。

一要保投资。全年安排固定资产投资1253亿元。其中，工业投资600亿元、城市建设投资380亿元、基础设施建设投资150亿元、农牧林及其它投资123亿元。为全市经济持续健康发展、各项事业稳步推进提供可靠保障。

二要保项目。市本级安排亿元以上重点项目216个。其中，工业类65个、城市建设类65个、基础设施类24个、农林水利类10个、商贸社会事业和民生类52个。这些重点项目是保投资的载体和抓手，要加大推进力度，确保按期开工建设。

三要保增量。目前，驰宏20万吨铅锌冶炼、宏裕科技“3052”化肥、荣达矿业4000吨/日矿石采选等一批重点项目已经具备了生产条件，要围绕这批项目，抓紧配套，解决问题，确保年内达产，形成增量拉动。

四要保运行。全力抓好经济运行的日常调度，落实好各项政策，从企业生产、物流和市场开拓等关键环节入手，解决企业实际困难。做好原料、融资等生产经营要素的组织和优化，保障企业平稳运行。特别要解决好停产、半停产企业的生产问题，挖掘、释放闲置生产能力，盘活存量。

（二）以“五大基地”建设为抓手，着力调整经济结构，转变发展方式

坚持以增量促结构调整，以创新转变发展方式，在发展中推动经济结构优化升级。

一要强力推进“五大基地”建设。建设清洁能源输出基地，推进大唐谢尔塔拉700万吨露天矿、宝日希勒1000万吨2号露天矿等项目建设，推动呼伦贝尔-山东±800千伏、呼伦贝尔-华北±660千伏电力外送通道和华能、神华等大型电力项目前期工作，抓好牙克石华能2×20万千瓦、扎兰屯华能2×35万千瓦和莫旗华电2×35万千瓦等热电联产项目。电力装机达到900万千瓦。建设现代煤化工生产示范基地，推进蒙东伊敏国家级现代煤化工基地规划，力争神华褐煤综合利用多联产升级示范项目开工，推动内蒙古矿业等煤化工项目前期工作。建设有色金属生产加工基地，确保驰宏20万吨铅锌冶炼项目投产，开工中金10万吨多金属矿分步回收综合利用项目，推进大兴安岭中段整装勘查，加快探转采步伐，尽快形成有色金属日采选矿石9万吨、年冶炼金属20万吨能力，逐步打造有色金属探、采、选、冶、加全产业链条。建设绿色农畜产品生产加工输出基地，依托农垦集团、阜丰、肉业集团、雨润及雀巢等骨干企业，提高农畜产品的转化率和精深加工水平，全年加工转化玉米300万吨、加工肉牛12万头、肉羊450万只。建设体现草原文化，独具北疆特色的旅游观光、休闲度假基地，把旅游业作为战略性支柱产业和结构调整的主攻方向，统筹布局，扎实推进，着力打造原生态、多民俗、国际性旅游目的地。

二要加快传统产业转型升级。木材加工产业，推动进口木材落地加工，支持满洲里木材加工园区做大做强。建材产业，加强技术改造，拉长产业链条，加快转型升级步伐。装备制造业，加快山东能源枣矿集团机械装备制造基地建设，今年要取得实质性进展。乳品产业，加强奶源基地建设，提升规模化、标准化养殖水平，扶持额尔古纳雀巢乳业发展。加大重组整合力度，优化岭东、岭西乳业布局，提高产业集中度。做好马铃薯、蓝莓、黑木耳、蘑菇等加工企业的整合提升。

三要培育壮大新兴产业。生物制药产业，确保鸿洋药业一期和北方药业二期投产，开工鸿洋药业二期和北方药业三期项目。信息产业，启动浪潮呼伦贝尔云计算项目运营，开工中网云计算中心项目。

四要加快工业园区建设。统筹推进“退二进三”和“退城进园”，实现企业向园区集中。加快自备电厂建设，推进大用户直供，完善基础设施配套，打造低成本优势。加快呼伦贝尔经济技术开发区、岭东工业园区、伊敏工业园区等重点园区建设。

（三）扭住农牧民增收这个核心，推进农牧业产业化

一要加大扶持力度。加大公共财政对农牧业的支持，整合各类专项资金，集中扶持龙头企业和农畜产品基地发展，支持农村牧区水利建设。注重科技支撑，提高农牧业科技成果转化应用水平。

二要继续调整种植结构。确保粮食增产增收，重点扩大玉米种植面积，今年达到1000万亩以上，玉米产量稳定在100亿斤以上。

三要进一步优化种养业结构。认真落实草原畜牧业三年转型规划和加快农区畜牧业发展的相关政策，草原畜牧业要稳定发展，农区畜牧业要加快发展，养殖业在第一产业中所占比重要有明显提升。

四要培育壮大龙头企业。着力培育现有产业化骨干企业，大力引进加工、流通等方面的大型龙头企业。争取年销售收入百万元以上的农畜产品加工企业达到230家，销售收入达430亿元以上。大力实施商标品牌战略，提升呼伦贝尔牛肉、羊肉、黑木耳和马铃薯四大品牌的影响力。

五要加快农畜产品基地建设。突出抓好肉牛、肉羊、乳业、生猪繁育基地建设和农田草牧场基础设施建设。新增各类规模化养殖场300个、人工饲草地35万亩，新增节水灌溉40万亩，其中玉米膜下滴灌30万亩，完成88万亩高标准农田整治任务。加快发展牧区生态家庭牧场。鼓励引导社会资本进入农牧业，建立基地与企业间有效利益联结机制，实现“共享阳光、共担风雨”。推进“粮安工程”，建设1.2万套农户科学储粮仓和4000吨应急低温成品粮储备库。推广高效肥料和低残留农药，构建农畜产品质量安全体系，发展生态农牧业。

（四）以旅游和物流产业为重点，加快服务业发展

一要全面推进旅游业。完成旅游发展总体规划，出台加快旅游业发展意见。抓好呼伦贝尔民族文化园、蒙古之源?蒙兀室韦民族文化园、拓拔鲜卑民族文化园、满洲里欧亚国际文化旅游度假区、扎兰屯国家级风景名胜区、大兴安岭北部原始森林国家公园等景区建设。推进旅游驿站建设和旅游纪念品开发。强化战略营销，着力开发冬季旅游、森林旅游和边境旅游。承办自治区蒙古族服饰大赛，举办呼伦贝尔大型摄影展以及冬夏那达慕等旅游活动。加强培训和监管，提升旅游服务水平。加快推动旅游业综合改革，筹建呼伦贝尔旅游集团。旅游接待人数和旅游业总收入均增长10%以上。

二要切实推进物流商贸产业。提升满洲里国际物流业，加快呼伦贝尔经济技术开发区工业物流和鄂温克汽车物流园建设，海拉尔中俄蒙国际物流园、牙克石大兴安国际物流园等重点物流园建设要有新突破。加快建设万达和红星美凯龙商业综合体，开工维多利商业综合体。推进万村千乡市场工程和新网工程。发展商务会展、电子商务、快递配送等各类新型消费业态。

三要加快培育养老健康产业。高度关注老龄化社会发展需求，出台扶持优待政策，引导社会资本投向养老健康产业，加快全市养老健康产业体系建设。年内要建设一批适应市场需求、功能完善的养老健康设施。

四要努力壮大金融业。加大对金融机构考评奖励力度，通过政府性资金存放管理，以存促贷。引导金融机构合理调整信贷方向，支持实体经济发展。加快发展地方金融机构和小额贷款公司，壮大融资性担保公司。创新投融资体制，鼓励支持金融机构、融资平台多渠道融资，扩大融资规模。

（五）强力推进新型城镇化进程，促进城乡协调发展

一要注重规划、突出特色。按照“以人为本、四化同步、科学布局、绿色发展、文化传承”的理念，依托我市独特的地域风光和民俗风情，科学编制和完善城镇总体规划和乡村规划，规划要显山、露水、留住根。要强调规划的权威性和刚性，做到一张蓝图绘到底，一届接着一届干。

二要统筹城乡发展。核心是发展县域经济，重点是培育支柱产业。各旗市区要从实际出发，确定和培育1-2个具有支撑作用的优势特色产业，以县域经济发展推动新型城镇化进程。

三要不断完善城市功能。实施240个市政基础设施建设项目，推进城市供热、供水、燃气、防洪排涝和污水垃圾处理等基础设施建设，开展城镇园林绿化和城市出入口、公路铁路沿线环境综合整治。支持民间资本进入市政公用事业领域。提升城镇管理水平，规范小区物业服务。

四要加大新区建设力度。加强中心城新区基础设施和公共服务配套建设，完善入托、入学、就医、餐饮、购物、休闲娱乐等设施和功能。强化中心城新区管理，以建筑工地和居民小区为重点，提升市容卫生水平，打造宜居宜业魅力新城。继续推进海拉尔组团、扎兰屯、牙克石等新区建设。

五要着力推进“十个全覆盖”。统筹城乡发展、推进城乡一体化，促进基础设施向农村牧区延伸，公共服务向农村牧区覆盖，城市文明向农村牧区辐射。我市村村通电、校舍建设及安全改造和便民连锁超市已有很好基础，今年抓好完善提高。农村牧区危房改造、安全饮水、嘎查村街巷硬化、农村牧区村村通广播电视通讯、嘎查村标准化卫生室、嘎查村文化活动室、农村牧区常住人口养老医疗低保社会保障等七项工作还存在很大差距。从今年起，要加强领导、科学规划、加大投入、决战3年，确保实现农村牧区“十个全覆盖”。

六要加快基础设施建设步伐。铁路投资31.8亿元，重点推进阿荣旗-莫旗、滨洲铁路电气化改造等12个项目。公路投资56亿元，重点推进国道那吉屯-尼尔基、省道根河-拉布大林等11条公路建设。民航投资3.7亿元，加快扎兰屯支线机场和4个通用机场建设，推动中国民航大学在扎兰屯设立飞行学校。电网投资20亿元，开工建设扎兰屯500千伏变电站工程，推进114项输变电工程建设。加快中心城老城区电网改造步伐。水利投资34.8亿元，重点推进Z866和扎敦水利枢纽建设，加快尼尔基水库下游30万亩灌区建设和晓奇子水利枢纽、诺敏阿兴灌区前期工作。继续完善平安城市和智能交通系统等信息基础设施建设，加快数字城市建设步伐，启动智慧城市试点工作。

（六）建设美丽呼伦贝尔，构筑北疆生态安全屏障

坚持不懈推进生态文明建设，做到老账加快还，新账不再欠，不留生态赤字、环境赤字。

一要抓源头。划定生态保护红线，落实国家主体功能区规划、《大小兴安岭生态保护与经济转型规划》和我市森林、草原、湿地等专项保护规划，实行最严格的源头保护制度。

二要抓过程。落实好退耕还草二期和草原生态保护补助奖励等各项政策，退牧还草480万亩、禁牧2851万亩、草畜平衡7508万亩。抓好退耕还林、天然林保护二期和重点公益林保护工程，森林采伐量控制在110万立方米以下。巩固森林城市创建成果，推进园林城市建设步伐，完成15万亩重点区域绿化，实施中心城区环城绿化等36项工程。治理沙地100万亩。做好呼伦湖综合治理工作，落实好为期5年的部分休渔措施。继续开展绿色矿山、和谐矿区建设，推进地质环境综合整治，对废弃矿坑、矿沟、探槽、采石场、取土场和挖沙点进行专项整治。凡新上项目坚决做到不符合主体功能区规划的不上、不符合环保要求的不上、不符合产业政策的不上。

三要抓结果。实施最严格的损害赔偿制度和责任追究制度，加大环境保护执法力度，严惩乱砍滥伐、毁林毁草开荒、草原挖药等行为。对违法排污企业严管重罚。加大节能减排力度，巩固生态建设成果。

（七）推进改革，扩大开放，增强发展的动力与活力

按照中央统一部署，扎实推进改革。一要深化农村牧区改革，加快构建新型农牧业经营体系，加大耕地草牧场流转力度，今年流转耕地720万亩以上、草牧场1500万亩。推进国有、集体土地使用权确权登记发证工作，抓好农村牧区“三资”清理后续监管和平台建设，探索建立农村牧区土地草牧场流转交易平台。二要成立国有资本管理机构和国有资本运营公司,实现对国有资产的有效监管。三要积极培育新的发展主体。筹建呼伦贝尔能源开发公司，组建呼伦贝尔地方森林经营公司，做好呼伦贝尔通用航空公司的运营执飞。四要深化政府机构改革，推进事业单位分类改革，探索建立新型科研机构运行机制和科技管理体制。五要进一步激发非公经济活力，落实好上级扶持非公经济发展的各项政策，发展混合所有制经济，鼓励民间资本参与国有企业改革。按照“非禁即入”的原则，下大决心降低准入门槛，千方百计优化发展环境，全心全意搞好服务，打造扶持非公经济发展的融资、担保、创业和服务平台。努力培育本土企业家，加快本土企业发展。全面落实国家相关政策，加快企业注册制度改革。

进一步加大对外开放力度，努力建设国家向北开放的桥头堡和充满活力的沿边经济带。全面深化与俄蒙毗邻地区的文化、经贸往来。推动满洲里开发开放试验区建设，加快满洲里综合保税区和自贸区申报工作。抓好《黑龙江和内蒙古东北部地区沿边开发开放规划》的实施。争取呼伦贝尔中俄蒙合作先导区方案尽快获得国家批复。推进中蒙跨境合作区建设，今年要有新突破。依托满洲里全国陆运指定粮食进口口岸的优势，加快绿色农畜产品落地加工。积极开拓广州、郑州等新的货源市场，建设经满洲里口岸的新铁路“丝绸之路”。外贸进出口总额和口岸过货量要有新提升。充分发挥自身优势，积极承接东北老工业基地、环渤海经济圈、长三角和珠三角等发达地区产业转移。

（八）持续保障和改善民生，全面推动社会事业发展

一要持续改善民生。进一步加大公共财政支出向民生和社会事业倾斜力度，全面落实各项惠民政策。实施“扶贫攻坚工程”，落实好金融扶贫和“金种子”扶贫等工程，整合项目资金，精准推进各类扶贫项目，实现全年减贫25%以上的目标。推进“创业就业工程”，建设创业就业服务平台和实训基地，实现高质量就业，全年城镇新增就业3.3万人，解决好高校毕业生和退役军人等重点人群的就业问题。抓好“百姓安居工程”，投资65亿元，开工建设各类保障性住房6.3万户。特别要抓好林区棚户区改造，按照“政府主导、企业主体、地企协作、共同推进”的原则，确保按时保质保量完成建设任务。继续实施“人才强市工程”，加大各类紧缺实用人才的培育和引进力度。提高社会保障能力，高度重视社会救助，完善临时救助和大病救助制度。提高企业退休人员养老金。城乡低保标准分别提高30元/月和300元/年，“五保”集中和分散供养标准分别提高到每年5100元和3200元。加快发展老龄事业，建设10个农村幸福院和20个社区老年人日间照料中心，爱心家园老年公寓投入使用，办好老年大学，实现全市80岁以上老人高龄补贴普惠制。继续推进“菜篮子”工程，加强反季节蔬菜和设施蔬菜基地建设，蔬菜种植面积达到20万亩，在中心城区建设10个大型储菜库和一批平价菜市场，千方百计稳定物价。解决15万人的饮水安全问题。推进中心城区集中供热工作，实施中心城区小烟囱治理。做好灾后救助和水毁工程修复的后续工作。

二要推动文化大市向文化强市转变。扎实开展以社会主义核心价值观为主要内容的群众性精神文明创建活动，提高公民道德素质和社会文明水平。加大非物质文化遗产保护力度，推进内蒙古蒙古族源博物馆建设，做好蒙古族源与元朝帝陵综合研究。深化国有文艺院团和文化事业单位改革，打造文化市场主体，开发文化演艺产品，推进文化产业发展进程，逐步实现文化大市向文化强市转变。

三要加快发展社会事业。启动呼伦贝尔科技创业服务中心，发挥科技孵化器培育功能，加强重点实用性技术推广应用。推进义务教育均衡协调发展，落实学前教育二期规划，加大民族教育和贫困地区教育基础设施建设力度，做好海拉尔一中迁建和海拉尔二中新区分校建设工作，做好中等专业学校资源整合和职业教育实训基地建设，提升高等院校办学水平。加强学校管理，抓好校车安全、校园安全和学生食品安全工作。推进医改示范区建设，完善卫生工作评价体系，激活基层卫生组织，加强卫生人才培养，扶持蒙中医药事业发展，扎实做好疾病预防控制工作，启动呼伦贝尔市医院新区分院建设，提升医疗救治水平。完成食品药品监管体制改革，加快食品安全全覆盖的监管体系和应急处置能力建设，保障人民群众饮食用药安全。加强人口计生工作，落实单独两孩生育政策。开展全民健身运动，加快15分钟健身圈场馆建设，提高体育竞技水平和群众健康水平。落实党的民族宗教政策，大力扶持少数民族各项事业，推动各民族共同团结奋斗、共同繁荣发展。发展公益慈善事业，保障残疾人权益。完成全国第三次经济普查工作，提升统计科学水平。加强行政监察和财政财务审计工作。提升防灾减灾和气象预警能力，强化质量监管，发展广电事业，统筹推进人防、地震和档案史志等工作。

（九）创新社会治理，建设北疆安全稳定屏障

继续推进农村牧区社区建设试点工作。以网格化管理、社会化服务为方向，提升社区服务能力与水平，及时反映和协调群众各方面各层次利益诉求。

激发社会组织活力。推进社会组织明确权责、依法自治、发挥作用。发展行业协会、商会，并引导其与行政机关、事业单位彻底分离。建立政府购买社会服务制度。

切实维护社会稳定。深入推进矛盾纠纷排查化解制度化、常态化，健全重大决策社会稳定风险评估机制，妥善解决群众合理诉求，把矛盾化解在源头。落实安全生产主体责任和党政同责、一岗双责，加大安全生产监督检查力度，保障人民群众生命财产安全。做好森林草原防火工作。继续实施“平安呼伦贝尔建设工程”，推动法治呼伦贝尔建设，加强社会治安综合治理，强化对网络舆情的规范管理，防范和打击各类违法犯罪活动，维护社会公平正义。加强国家安全教育，推进国防动员和军警民边防体系建设，力争国家级双拥模范城创建实现“四连冠”。

认真执行人大及其常委会各项决议、决定，自觉接受监督。积极支持政协履行各项职能。认真办理人大代表议案、建议和政协委员提案。主动听取各民主党派、工商联、无党派人士及各人民团体的意见和建议。支持工会、共青团、妇联等群团组织按照各自职能开展工作。

（十）努力建设人民满意政府

中央决定今年开展第二批党的群众路线教育实践活动。我们要以此为契机，全面加强政府自身建设，切实做到为民、务实、廉洁。一要建设为民政府。我们的政府是人民政府，由人民选举、对人民负责、为人民服务，这是我们要始终牢记的宗旨。要忠于人民，时刻把人民群众的需求、意愿作为政府施政的第一信号。要敬畏人民，以谦卑之心对待人民群众，深入实际，调查研究，时刻关心群众生活，切实把群众住房、医疗、交通、上学、供暖、供水、供电以及柴米油盐等问题解决好。二要建设务实政府。抓落实是政府的主要职责，坚决克服形式主义和官僚主义，做到说了就办，定了就干，切实提高政府的公信力和执行力。要敢于担当、勇于负责，切实提高政府工作效率。推进政务公开和应急管理工作。继续简政放权，巩固行政审批制度改革成果，启用市本级政务服务中心和公共资源交易中心，所有审批事项要在服务中心办理，所有公共资源集中进行交易。三要建设廉洁政府。要认真贯彻落实中央八项规定、自治区28项具体要求和市委有关要求，严格遵守党风廉政建设各项规定。要加大源头治腐力度，扎实推进惩治和预防腐败体系建设。依法履行职能，坚持用制度管人、管钱、管事，凡是重大事项决策、重要项目安排和大额度资金使用必须按照规定执行，让权力在阳光下运行。认真落实国务院“约法三章”，做到政府性楼堂馆所一律不得新建，财政供养人员只减不增，公务接待、公费出国和公费购车严格执行上级规定。继续清理超标准办公用房和超标超编公务用车。加强政府采购、资源配置等重点领域的制度建设，强化建设领域的招投标和专项资金的规范化使用，把权力关进制度的笼子里。

各位代表，今年经济社会发展的目标任务已经确定。让我们在自治区党委、政府和市委的坚强领导下，团结带领全市各族人民，守望相助、和衷共济，为建设幸福呼伦贝尔而努力奋斗！
